# The effect of pegylated interferon-alpha2b and ribavirin combination therapy for chronic hepatitis C infection in elderly patients

**DOI:** 10.1186/1756-0500-5-135

**Published:** 2012-03-10

**Authors:** Hiroki Nishikawa, Eriko Iguchi, Yorimitsu Koshikawa, Soichiro Ako, Tadashi Inuzuka, Haruhiko Takeda, Jun Nakajima, Fumihiro Matsuda, Azusa Sakamoto, Sinichiro Henmi, Keiichi Hatamaru, Tetsuro Ishikawa, Sumio Saito, Ryuichi Kita, Toru Kimura, Yukio Osaki

**Affiliations:** 1 Department of Gastroenterology and Hepatology, Osaka Red Cross Hospital, Osaka, Japan; 2 Department of Gastroenterology and Hepatology, Osaka Red Cross Hospital, 5-30 Fudegasaki-cho, Tennoji-ku, Osaka 543-0027, Japan

**Keywords:** Chronic hepatitis C, Pegylated interferon, Ribavirin, Treatment response, Elderly patients

## Abstract

**Background:**

The clearance of hepatitis C virus infection by interferon therapy significantly reduces the incidence of hepatocellular carcinoma and death in elderly chronic hepatitis patients. However, there are few reports concerning the efficacy and safety of pegylated interferon-alpha2b plus ribavirin combination therapy in elderly patients. The aims of the present study were to examine the effect and safety of pegylated interferon-alpha2b plus ribavirin combination therapy in 427 patients with chronic hepatitis C infection. We compared the rates of sustained virological response--defined as the absence of detectable hepatitis C virus in serum 24 weeks after the treatment ended--and the treatment discontinuation rate between 319 younger patients aged < 65 years and 108 elderly patients aged ≥ 65 years. We also examined the factors contributing to a sustained virological response.

**Results:**

There was no significant difference in the sustained virological response rate between younger patients and elderly patients according to their hepatitis C virus genotype (41.5% (100/241) and 40.7% (35/86) for genotype 1; *P *= 0.899, 89.7% (70/78) and 86.4% (19/22) for genotype 2; *P *= 0.703, respectively). There was also no significant difference in the treatment discontinuation rate between the two age groups (10.3% (33/319) and 13.9% (15/108), respectively; *P *= 0.378). There were no serious adverse events requiring hospitalization. The factors contributing significantly to a sustained virological response in elderly patients were gender, hepatitis C virus genotype, platelet count, and the presence of a rapid or early virological response (undetectable hepatitis C virus in serum at weeks 4 or 12 of treatment, respectively). However, upon multivariate analysis, the presence of an early virological response was the only significant factor (odds ratio: 0.115, 95% confidence interval: 0.040- 0.330, *P *< 0.001).

**Conclusions:**

The efficacy and safety of pegylated interferon-alpha2b plus ribavirin combination therapy in elderly patients are not always inferior to those in younger patients. Obtaining an early virological response may be essential to achieve a sustained virological response in elderly patients with chronic hepatitis C infection.

## Background

Chronic hepatitis C virus (HCV) infection affects approximately 300 million people worldwide and is the most common cause of chronic liver disease [1]. Among HCV-infected individuals, 20-30% eventually develop cirrhosis or hepatocellular carcinoma (HCC). In Japan, approximately 30,000 people die of HCC every year, and 70-80% of these deaths are because of HCV infection. Therefore, it is important to eliminate HCV with interferon (IFN) therapy to prevent HCC [2,3].

Hepatitis C virus entered and expanded in Japan decades before it did so in the United States [4]. In Japan, the patients with chronic HCV who are currently treated with IFN are 10 to 15 years older than the corresponding patients in the United States and Western countries, where the patients treated with antiviral therapy average 45 years of age.

In Japan, HCV infection is characterized by an increasing prevalence with age [5]. Previous studies have reported that clearance of HCV by IFN therapy significantly reduces the incidence of HCC and death in older chronic hepatitis patients [3]. Therefore, it is particularly important to eliminate HCV in elderly patients.

The standard treatment for patients chronically infected with HCV is pegylated interferon (Peg-IFN) and ribavirin combination therapy for 24 or 48 weeks, according to the HCV genotype [6]. This treatment protocol reportedly obtained sustained virological response (SVR) rates-defined as undetectable HCV in serum 24 weeks after the treatment ended--ranging from 45% in patients with HCV genotype 1 to 85% in patients with genotypes 2 or 3 [7,8]. Recently, the addition of a protease inhibitor to Peg-IFN plus ribavirin combination therapy (triple therapy) has been reported to improve the anti-viral effect [9]. However, these figures were obtained in patients aged 40 to 50 years, as older patients have been excluded from large therapeutic trials [7-9]. Therefore, to date, there are few data concerning the response and safety of this combination treatment in elderly patients [10-12].

The aims of the present study were to examine the effect and safety of Peg-IFN-alpha2b and ribavirin combination therapy in elderly patients and to examine the factors contributing to the SVR in this combination therapy.

## Methods

### Patients

Between January 2005 and April 2008, 427 consecutive patients with chronic hepatitis C were treated with Peg-IFN-alpha2b and ribavirin combination therapy at the Department of Gastroenterology and Hepatology, Osaka Red Cross Hospital, Japan. Each patient was followed up for at least 24 weeks after the treatment ended. Of the 427 patients, 319 were < 65 year old (younger patients) and 108 were ≥ 65 years old (elderly patients). Whether the patients were treated with this combination therapy was mainly based on the decisions of their attending physicians, considering factors such as age, motivation for the combination therapy, general condition, and underlying diseases. Treatment of chronic HCV with a combination of Peg-IFN-alpha2b and ribavirin was approved by the Japanese Ministry of Health in October, 2004. However, the use of PEG-IFN-alpha2a and ribavirin was only approved in September, 2008. Therefore, in the present study, all patients were treated with PEG-IFN-alpha2b and ribavirin combination therapy.

We analyzed the clinical data retrospectively. Patients were excluded if they tested positive for human immunodeficiency virus infection, or if they had decompensated liver disease, liver disease of other cause, psychiatric illness, severe cardiovascular disease, autoimmune disease, poorly controlled diabetes mellitus, renal disease, or alcohol abuse. A liver biopsy was performed in all patients before the treatment, and all biopsy specimens were assigned a fibrosis score and an activity score based on the criteria of Bedossa et al. [13]. This study was conducted according to the ethical guidelines of the 1975 Declaration of Helsinki, and informed consent was obtained from all patients. We received ethical approval for the present study from the ethics committee of our hospital, Osaka Red Cross Hospital, Japan.

### Treatment schedule

Patients were treated with Peg-IFN-alpha2b (1.5 μg/kg/week) plus oral ribavirin according to the Japanese guidelines (total dose 600 mg/day for patients weighing < 60 kg; 800 mg/day for patients with a body weight of 60-80 kg; 1,000 mg/day for patients weighing > 80 kg). Treatment was given for 48 weeks for genotype 1 and 24 weeks for genotype 2. If hematologic side effects were observed, the doses were reduced according to information from the manufacturer. However, in the case of side effects associated with subjective symptoms of Grade 2 or above, such as malaise, fever, anorexia, or light-headedness, the dose of Peg-IFN was reduced by 10-20 μg/week, and the dose of ribavirin was reduced by 200 mg/day as soon as possible, and both were maintained at the lower levels until the severity of the symptoms lessened to Grade 1 or below. During the treatment, we did not use erythropoietin or granulocyte-macrophage colony stimulating factor in any patient.

### Virological evaluation

Serum HCV-RNA levels were quantified and qualitatively analyzed using a Cobas Amplicor HCV Monitor Test, version 2.0 (detection range 6-5,000 kIU/ml, lower limit of detection 50 IU/ml; Roche Diagnostics, Branchburg, NJ). HCV genotype was determined by polymerase chain reaction (PCR) amplification of the core region of the HCV genome using genotype-specific PCR primers. [14]

### Assessment of treatment efficacy

A rapid virological response (RVR) and an early virological response (EVR) were defined as undetectable serum HCV-RNA at week 4 and week 12 of the therapy, respectively. An end-of-treatment response was defined as undetectable HCV-RNA at the end of the therapy (*i.e*., week 48 for genotype 1, week 24 for genotype 2). A sustained virological response (SVR) was defined as undetectable HCV-RNA 24 weeks after completion of treatment. All assessment methods were defined according to published guidelines [15,16]. Even if the treatment was prematurely discontinued because of side effects or non-compliance, patients could be considered to have an SVR on the basis of their serum HCV-RNA results at the 24-week follow-up. Non-response was defined as a lack of HCV-RNA clearance during treatment and at follow-up. During the follow-up period, clinical, biochemical, and qualitative serum HCV-RNA evaluations were performed every 3 months.

### Statistical analysis

Variables were compared between groups by the chi-squared test, Fisher's exact probability test, and the Mann-Whitney U test. Group means, presented as mean values ± standard deviations, were compared using the Student's *t*-test. The influence of various factors on the response to Peg-IFN and ribavirin combination therapy was evaluated by univariate analysis. All variables found to be significant in the univariate analysis were subjected to multivariate analysis using logistic regression. The data were analyzed using SPSS version 9.0 for Microsoft Windows (SPSS, Inc., Chicago, IL). All statistical analyses were based on two-sided hypothesis tests with a significance level of *P *< 0.05.

## Results

### Patients

The baseline characteristics of the elderly patients enrolled in the present study are shown in Table 1. The number of genotype 1 and 2 patients was 86 (79.6%) and 22 (20.4%), respectively. No patients with HCV genotype 3 or 4 were included in the present study because there are very few patients with these genotypes in Japan. All the patients had a high viral load (≥ 10 klU/ml), according to the Japanese criteria. The number of patients aged ≥ 70 years was 24 (22.2%). The oldest patient was an 81-year-old male, and he achieved an SVR.

**Table 1:** Baseline characteristics of patients aged ≥ 65 years (*n *= 108)

Category	*n *or mean ± SD
Gender (Male/Female)	54/54

Age	68.2 ± 2.8

Body Weight (kg)	58.4 ± 9.5

Body mass index (kg/m^2^)	23.3 ± 3.0

History of interferon therapy (yes/no)	40/68

History of diabetes mellitus (yes/no)	19/89

History of hypertension (yes/no)	47/61

White blood cells (/mm^3^)	4909 ± 1332

Red blood cells (× 10^4^/mm^3^)	436.1 ± 39.8

Hemoglobin (g/dL)	13.8 ± 1.2

Platelets (× 10^4^/mm^3^)	15.3 ± 3.9

AST (IU/L)	65.2 ± 37.3

ALT (IU/L)	76.0 ± 65.5

AFP (ng/ml)	15.7 ± 31.4

Histology (METAVIR) Fibrosis (1/2/3/4)	22/41/37/8

Histology (METAVIR) Activity (1/2/3)	30/51/27

HCV genotype (1/2)	86/22

Serum HCV RNA (KIU/ml)	1650 ± 1521

Peg-IFN dose (g/kg/week)	1.22 ± 0.32

Total Peg-IFN dose > 80% (yes/no)	63/45

Daily ribavirin dose (mg/kg)	9.58 ± 2.93

Total ribavirin dose > 80% (yes/no)	63/45

Data are shown as absolute values or mean ± standard deviation. *AST *aspartate aminotransferase; *ALT *alanine aminotransferase; *AFP *alpha-fetoprotein; *HCV *hepatitis C virus; *Peg-IFN *pegylated interferon

### Assessment of treatment response

SVR rates in the younger patients and the elderly patients according to their HCV genotype were 41.5% (100/241) and 40.7% (35/86) for genotype 1 and 89.7% (70/78) and 86.4% (19/22) for genotype 2, respectively. In terms of SVR rates, there were no significant differences between the two age groups according to HCV genotype (*P *= 0.899 for genotype 1 and *P *= 0.703 for genotype 2, respectively) (Table 2).

**Table 2:** SVR rates between patients aged < 65 years and patients aged ≥ 65 years according to HCV genotype

	Age < 65 (*n *= 319)	Age ≥ 65 (*n *= 108)	*P *value^a^
SVR rate (Genotype 1)	41.5% (100/241)	40.7% (35/86)	0.899

SVR rate (Genotype 2)	89.7% (70/78)	86.4% (19/22)	0.703

*HCV *hepatitis C virus; *SVR *sustained virological response; ^a ^Fisher's exact test

Among the elderly patients, the overall SVR rate was 50% (54/108). An RVR was obtained in 24 patients (10 of genotype 1, 14 of genotype 2), an EVR was obtained in 61 patients (41 of genotype 1, 20 of genotype 2), and an end-of-treatment response was obtained in 89 patients (68 of genotype 1, 21 of genotype 2). The number of patients with a non-response was 19 (18 of genotype 1, 1 of genotype 2). All the patients who demonstrated an RVR achieved an SVR, and 47 (77%) of the 61 patients who obtained an EVR achieved an SVR. Among the 47 patients who did not obtain an EVR, only seven (14.9%) achieved an SVR. According to gender, 33 males (61.1%) and 21 females (38.9%) achieved an SVR. Among the 63 patients who completed at least 80% of the scheduled Peg-IFN dose, 36 (57.1%) achieved an SVR; similarly, among the 63 patients who completed at least 80% of the scheduled ribavirin dose, 36 (57.1%) achieved an SVR. Among the 47 patients who completed at least 80% of the scheduled Peg-IFN-alpha2b dose, ribavirin dose, and scheduled treatment duration (80/80/80 adherence, as mentioned elsewhere [17]), 27 (57.4%) demonstrated an SVR.

### Factors contributing to SVR in elderly patients

In our univariate analysis of factors contributing to an SVR in elderly patients, gender (*P *= 0.021), HCV genotype (1 or 2) (*P *< 0.001), platelet count (*P *= 0.003), the presence of an RVR (*P *< 0.001), and the presence of an EVR (*P *< 0.001) contributed significantly (Table 3). However, in our multivariate analysis of these five factors by logistic regression, the presence of an EVR was the only significant factor (odds ratio: 0.115, 95% confidence interval: 0.040-0.330, *P *< 0.001) (Table 4).

**Table 3:** Univariate analysis of factors contributing to an SVR in elderly patients (*n *= 108)

Risk factor	SVR (*n *= 54)	non SVR (*n *= 54)	*P *value
Gender (male/female)	33/21	21/33	0.021

HCV genotype, (1/2)	35/19	51/3	< 0.001

Previous IFN therapy, (yes/no)	16/38	24/30	0.163

Diabetes mellitus, (yes/no)	7/47	12/42	0.312

Hypertension, (yes/no)	22/32	25/29	0.698

Body mass index (kg/m^2^), mean ± SD	23.0 ± 3.0	23.7 ± 3.0	0.212

White blood cells (/mm^3^), mean ± SD	4948 ± 1412	4869 ± 1260	0.761

Hemoglobin (g/dL), mean ± SD	13.9 ± 1.2	13.8 ± 1.2	0.583

Platelets (× 10^4^/mm^3^), mean ± SD	16.4 ± 4.2	14.2 ± 3.3	0.003

AST (IU/L), mean ± SD	69.1 ± 44.0	61.3 ± 29.2	0.280

ALT (IU/L), mean ± SD	86.9 ± 80.7	65.1 ± 43.7	0.084

AFP (ng/ml), mean ± SD	12.1 ± 19.6	19.4 ± 39.7	0.228

Fibrosis score, (F1,2/F3,4)	36/18	27/27	0.079

Serum HCV RNA (KIU/ml), mean ± SD	1290 ± 1441	1343 ± 1265	0.838

Peg-IFN does (μg/kg week), mean ± SD	1.23 ± 0.31	1.21 ± 0.30	0.494

Daily ribavirin dose (mg/kg), mean ± SD	9.68 ± 2.96	9.48 ± 2.92	0.268

80/80/80 adherence, (yes/no)	27/27	20/34	0.244

rapid virological response, (yes/no)	24/30	0/54	< 0.001

early virological response, (yes/no)	47/7	14/40	< 0.001

Data are shown as absolute values or mean ± standard deviation. *AST *aspartate aminotransferase; *ALT *alanine aminotransferase; *AFP *alpha-fetoprotein; *HCV *hepatitis C virus; *Peg-IFN *pegylated interferon; *SVR *sustained virological response

**Table 4:** Multivariate analysis of factors contributing to an SVR in elderly patients

Risk factor		Odds ratio	95% confidence interval	*P *value
RVR				

	yes	1		0.998

	no	0.000	0.000	

EVR				

	yes	1		

	no	0.115	0.040-0.330	< 0.001

HCV genotype				

	1	0.534	0.093-3.050	0.480

	2	1		

Gender				

	male	1.055	0.370-3.004	0.921

	female	1		

Platelet count				

	> 15 × 10^4^/mm^3^	1		

	≤ 15 × 10^4^/mm^3^	0.838	0.285-2.468	0.748

*RVR *rapid virological response; *EVR *early virological response; *HCV *hepatitis C virus

### Tolerability and adverse events

There was no significant difference in the treatment discontinuation rate between younger patients and elderly patients (10.3% (33/319) and 13.9% (15/108), respectively; *P *= 0.378) (Table 5). The causes of treatment discontinuation are shown in Table 5. In eight younger patients and four elderly patients, the attending physician decided to discontinue treatment because the viral load had not decreased by week 24 of the treatment. There were no adverse events that required hospitalization.

**Table 5:** Causes of treatment discontinuation for the two age groups

	Aged < 65 years group(*n *= 33)	Aged ≥ 65 years group(*n *= 15)
Anemia	9	5

Thrombopenia	9	3

General fatigue	7	3

Poor response to therapy	8	4

## Discussion

In ageing chronic hepatitis C patients, especially those over 60 years, liver fibrosis is accelerated and the incidence of HCC is increased [18,19]. Among aged patients, improving the liver fibrosis and decreasing the risk of HCC are mainly achievable by eradication of the hepatitis C virus [2]. Therefore, the primary goal of the IFN therapy in elderly patients should be HCV eradication.

In the present study, there was no significant difference in SVR rate between the two age groups according to HCV genotype. Moreover, there was no significant difference in the treatment discontinuation rate between the two age groups. Our data suggest that Peg-IFN-alpha2b and ribavirin combination therapy is just as tolerable and effective in elderly patients as in younger patients.

The SVR rate and the discontinuation rate of patients aged ≥ 65 years in the present study are better than those of previous studies [20-24]. Oze et al. reported that the Peg-IFN dose affects the virologic response [25], and Hiramatsu et al. reported that ribavirin dose reduction increases the relapse rate [26]. However, in the present study, there were no significant relationships between the total Peg-IFN dose, total ribavirin dose, or treatment duration and SVR. These differences may have arisen because the previous studies were conducted mainly in patients aged ≤ 65 years. In the case of elderly patients, maintaining drug therapy at the standard dose often causes side effects, which makes treatment discontinuation or significant treatment dose reduction inevitable. Oze et al. [24] investigated the efficacy and adverse effects of Peg-IFN plus ribavirin therapy in aged patients with chronic hepatitis C. We compared their SVR and treatment discontinuation rates with ours according to the dose of Peg-IFN and ribavirin. Although Oze et al. [24] used a larger dose of both Peg-IFN and ribavirin than we did (Peg-IFN dose: 1.22 ± 0.32 μg/kg/week (our data) *vs*. 1.44 ± 0.18 μg/kg/week (Oze et al.); ribavirin dose: 9.58 ± 2.93 mg/kg/day (our data) *vs*. 11.5 ± 1.7 mg/kg/day (Oze et al.)), the SVR rate was 40.7% in genotype 1 elderly patients in our study and less than 30% in genotype 1 elderly patients in the study by Oze et al. The treatment discontinuation rate in elderly patients was also significantly different between the studies: 13.9% in our study *vs*. less than 30% in the study by Oze et al. A recent large-scale study demonstrated that the SVR rate did not differ between standard-dose (1.5 μg/kg/week) and low-dose (1.0 μg/kg/week) Peg-IFN-alpha2b therapy [27]. In fact, in elderly patients, dose reduction in a relatively early phase, based on careful monitoring of side effects, may prevent treatment discontinuation and lead to an improved SVR rate.

Sezaki et al. reported that in elderly patients, the response to Peg-IFN and ribavirin combination therapy is worse in female than in male patients [28]. Our data are in agreement with this (SVR of 61.1% in males and 38.9% in females; *P *= 0.021).

In our multivariate analysis, the presence of an EVR was the only factor contributing significantly to an SVR. Although the positive predictive value for an RVR was 100% (24/24), it was not a significant factor in the multivariate analysis probably because an SVR was achieved without an RVR in many cases. The key to successful IFN therapy in elderly patients is likely to be achieving an EVR. However, if this requires maintaining a high Peg-IFN dose, there is a high possibility that subsequent large dose reduction or even treatment discontinuation will be needed. Thus, careful monitoring is necessary in elderly patients, especially within the first 12 weeks of their treatment. Moreover, if an EVR is not achieved, early discontinuation of treatment may be important in terms of preventing unnecessary treatment and side effects, because, in the present study, among the 47 patients who did not obtain an EVR, only seven (14.9%) achieved an SVR.

This study has several limitations. First, our study is a retrospective study. Second, the number of elderly patients in the present study was low. In our multivariate analysis, the presence of an EVR was the only significant factor contributing to an SVR in elderly patients. However, in general, the four factors that were significant in our univariate analysis (*i.e*., the presence of an RVR, gender, HCV genotype, and platelet count) are also reported to be significant predictive factors for an SVR [21-26]. Therefore, we should interpret our results with caution, and larger studies are needed. Third, all patients who had serious underlying diseases were excluded from our study. In elderly patients with serious underlying diseases, an ursodeoxycholic acid and/or glycyrrhizin-containing preparation (Stronger Neo-Minophagen C) will be needed when serum alanine aminotransferase levels are higher than the normal upper limit, because these patients would be expected to experience serious side effects from Peg-IFN-alpha2b plus ribavirin combination therapy or other IFN therapy.

Therefore, it is disputable whether the results of our study are applicable to all elderly patients, who often have underlying diseases. These issues should be clarified in future prospective studies.

## Conclusion

In conclusion, the effects of Peg-IFN-alpha2b plus ribavirin combination therapy for HCV infection in elderly patients are not always inferior to those in younger patients. Peg-IFN-alpha2b plus ribavirin combination therapy may be safely extended to elderly patients who have no serious underlying disease.

## Competing interests

The authors declare that they have no competing interests. None of the authors has had within the past 12 months any financial relationship with a biotechnology manufacturer, a pharmaceutical company, or other commercial entity that has any interest in the subject matter, materials, or processes discussed in the manuscript.

## Authors' contributions

YO, TK, SS, AS, and TI participated in the design of the study and performed the statistical analysis; RK helped to draft the manuscript. EI, SA, YK, TI, HT, FM, JN, KH, and SH collected the data. All authors have read and approved the final manuscript.
